# Functional Constipation and the Gut Microbiome in Children: Preclinical and Clinical Evidence

**DOI:** 10.3389/fped.2020.595531

**Published:** 2021-01-20

**Authors:** David Avelar Rodriguez, Jelena Popov, Elyanne M. Ratcliffe, Erick Manuel Toro Monjaraz

**Affiliations:** ^1^Department of Pediatric Gastroenterology and Nutrition, Instituto Nacional de Pediatría, Mexico City, Mexico; ^2^Department of Pediatrics, The Hospital for Sick Children, Toronto, ON, Canada; ^3^College of Medicine and Health, University College Cork, Cork, Ireland; ^4^Division of Gastroenterology and Nutrition, Department of Pediatrics, McMaster University, Hamilton, ON, Canada

**Keywords:** functional constipation, childhood constipation, gut microbiota, gut microbiome, functional gastrointestinal disorders

## Abstract

Functional constipation is a common condition in childhood with significant impact on patients' quality of life and on health care resources. Functional constipation is characterized by decreased bowel movements and/or hard stools, which cause significant distress for children and their caregivers. While the term “functional” may imply the absence of organic causes with a focus on behavioral aspects, 40% of children continue to have symptoms beyond conventional management with one in four children continuing to experience constipation into adulthood. The refractory and chronic nature of constipation highlights the importance of considering a range of pathophysiological mechanisms, including the potential role of the gut microbiome. In this review, we provide an overview of preclinical and clinical studies that focus on the potential mechanisms through which the gut microbiome might contribute to the clinical presentation of functional constipation in pediatrics.

## Introduction

Functional constipation (FC) is a common condition in childhood (1) which causes significant distress and impairs the quality of life of children and parents (2). FC also causes a significant burden on healthcare resources through the frequent use of costly medical services (3, 4). FC is characterized by decreased bowel movements and/or hard stools and can be complicated by retentive fecal incontinence (5). FC is a clinical entity that can be diagnosed based on the child's bowel habits, signs and symptoms, and according to the Rome IV criteria (6–8). In equivocal cases or the presence of red flags, ancillary investigations can be used to rule out organic causes such as Hirschsprung's disease, anorectal malformations, pediatric intestinal pseudo-obstruction, hypothyroidism, and celiac disease (8).

The term “functional” may imply the absence of an organic etiology and emphasizes behavioral factors (see below) as the main drivers of the disease (6, 7). However, the fact that approximately 25% of children continue to experience symptoms in adulthood (9) and only 60% of children are symptom-free after 6–12 months, (10) suggests the involvement of other pathophysiological mechanisms. Non-adherence to pharmacotherapy has previously been cited as one important factor contributing to poor outcomes (11). More recently, the gut microbiome has begun gaining increasing attention in guidelines (8) and expert reviews (12, 13). However, there is seldom elaboration on how the gut microbiome may contribute to the pathophysiology of pediatric FC as the current evidence is limited and often contradictory. Therefore, this state-of-the-art review aims to explore and summarize the possible mechanisms of interaction between the gut microbiome and FC in children, based on data from preclinical and clinical studies.

## Epidemiology and Associated Factors

The true prevalence of FC in children is difficult to determine due to the important limitations of study methodologies, such as population sampling, study setting, data collection, and study homogeneity (14, 15). The most recent systematic reviews and meta-analysis (1) have estimated a global pooled prevalence of 9.5% (0.5–32%), with significantly more affected children in America and Europe than in Asia. These findings differ from a prior systematic review which reported a mean prevalence of 16% (16). A recent observational study of >1,100 Sri Lankan children reported a prevalence of 8% and increased risk for children living in urban areas and children who are underweight for their age (17). FC in children does not appear to have a sex or age predilection (1, 15, 17).

Certain factors have been associated with an increased risk of developing FC. Childhood traumas such as peer bullying and verbal/physical abuse within the family have been suggested (1, 18, 19), however the evidence is contradictory (20). Children with difficult temperaments, emotional/behavioral problems, and frequent temper tantrums may also be more likely to experience constipation and soiling (21). Socioeconomic status does not appear to be a significant factor (1, 17, 20). However, dietary habits and physical activity may play an important role as children with FC report lower intake of fruits and vegetables, increased consumption of fast foods, and lower physical activity levels (1). At present, the true cause-effect nature of these interactions remains unclear due to the designs of observational studies and their inherent risk of bias.

## Pathophysiology

The pathophysiology of FC is multifactorial, involving a complex interplay between behavioral factors and alterations in the gut microbiome and gastrointestinal motility.

### Behavioral Aspects

The trigger for FC is often the painful passage of hard stools, which causes the child to enter a vicious cycle of withholding stools and thus worsening constipation (6, 7). In a recent systematic review (22), 17 unique behaviors were described to be associated with functional defecation disorders, including behaviors related to fear or anxiety concerning defecation or sitting on the toilet. The most commonly reported behavior was described as “stool toileting refusal,” which is a likely contributor to the vicious cycle of withholding behaviors (22).

### The Gut Microbiome

The gut microbiome is composed of trillions of microorganisms and is considered the “body's virtual organ” given its importance in maintaining host homeostasis (23). Disruptions in the gut microbiome are often referred to as “dysbiosis” and have increasingly been associated with a number of disease states in children (24), including functional gastrointestinal disorders such as irritable bowel syndrome (25–27) and constipation (28–30).

#### Bacterial Colonization in Early Life

Neonatal and infancy periods are strongly influenced by the mode of delivery and feeding (23). Infants born via vaginal delivery exhibit microbiome profiles resembling the maternal vaginal flora, dominated by *Lactobacillus, Prevotella*, or *Sneathia* spp, indicating direct vertical transmission. In contrast, cesarean section infants exhibit higher levels of *Staphylococcus, Corynebacterium*, and *Propionibacterium* spp. (31), which are common components of skin flora. While formula-fed infants harbor a wider diversity of bacteria, breastfed infants exhibit greater interactions between microbial and host genes, indicating higher levels of bioactive compounds in breastmilk compared to formula milk (32). Furthermore, in contrast to the traditional belief that the introduction of solid-foods drives maturation of the infant microbiome, recent evidence suggests that it may be the cessation of breastfeeding which has the greatest impact on promoting microbial community shifting to resemble an adult phenotype (33). Interestingly, children with FC are more likely to have a history of delivery via cesarean section and a shorter duration of breastfeeding compared to controls with no history of constipation (29). This suggests that early gut microbiome insults [e.g., cesarean delivery, antibiotic use, formula-feeding/shorter duration of breastfeeding, or intrapartum antibiotic administration (34)] may increase the risk of developing FC later in life. For instance, children with a history of necrotizing enterocolitis, most of whom are premature and are virtually all treated with prolonged courses of antibiotics, have a higher incidence of FC compared to healthy children (35).

#### Evidence From Animal Models

Preclinical data from germ-free (GF) and antibiotic-treated animal models have supported the role of the gut microbiome in regulating gastrointestinal motility through complex neuroendocrine and metabolic mechanisms. Microbial-derived metabolites such as tryptamine (36) and short-chain fatty acids (SCFAs), (37, 38) have been shown to promote intestinal motility. Tryptamine, a monoamine with a similar structure to serotonin (5-HT), was shown to increase anion and fluid secretion and stimulate whole-gut transit through activation of the 5-HT4 receptor, suggesting therapeutic potential (36). In another study (38), GF mice were found to have abnormal colonic peristalsis characterized by non-propulsive motility, which was normalized following butyrate administration. Conversely, butyrate did not affect the intestinal contractions of tryptophan hydroxylase-1 (TPH1) knockout (KO) mice (i.e., mice lacking mucosal 5-HT), suggesting that its effect may require mucosal 5-HT. Furthermore, propionate was shown to inhibit propulsive contractions in all mice. Gut microbiota may also modify gastrointestinal motility by suppressing the expression of glucagon-like peptide 1 (GLP-1), although the mechanism through which this occurs has yet to be elucidated (39).

Interestingly, a recent investigatory study (40) assessing the effects of *Lactobacillus rhamnosus* on treating loperamide-induced constipation showed variable degrees of symptomatic improvement which were bacterial strain-specific and independent of the previously reported SCFA effects (37, 38). Specifically, *L. rhamnosus* increased serum peptide YY, colonic 5-HT, colonic brain-derived neurotrophic factor, and significantly increased α-diversity, with minor effects on improving β-diversity. Another recent study (41) also used a loperamide-induced constipation mouse model and found that organisms from human breastmilk such as *Pediococcus pentosaceus* B49 may contribute to marked improvements in intestinal transit time and stool production. Supplementation with *P. pentosaceus* B49 was also associated with significant reductions in pathogenic bacteria at the genus level (*Alloprevotella, Staphylococcus*, and *Helicobacter*), and normalization of expression levels of genes involved in gastrointestinal water and ion transport, peristalsis, inflammation, and even malignancies.

Studies in which the microbiome has been perturbed by antibiotics suggest a potential link to intestinal motility. Mice receiving 4 weeks of antibiotic treatment were found to exhibit overall lower intestinal and colonic transit times, as well as decreased levels of lithocholic acid and microbial-derived 5-HT production compared to controls (42). While muscle tension was significantly decreased in the antibiotic-treated group, the frequency of intestinal contractions remained unchanged. Another study assessing antibiotic-induced dysbiosis in juvenile demonstrated similar effects, including delayed gastrointestinal transit, reduced frequency of stool expulsion, and altered intestinal smooth muscle contractility (43).

Perturbations in the gut microbiome might influence intestinal motility through alterations in the enteric nervous system (ENS). Mice with an intact gut microbiome exhibit increased expression of the transcription factor aryl hydrocarbon receptor (AHR) in colonic neurons compared to antibiotic-treated mice and GF mice, whose intestinal peristaltic activity is reduced (44). Interestingly, AHR promotes intestinal peristalsis, particularly in the colon, through a bidirectional interaction with the gut microbiome. Moreover, antibiotic-induced dysbiosis can cause significant decreases in cholinergic neurons and overall myenteric neurons, and distortions in glial networks within the myenteric plexus (43). While antibiotic-induced intestinal dysbiosis does not appear to alter the frequency of contractions, an absence of gut microbiota in the perinatal period may affect smooth muscle function. Specifically, GF and altered Schaedler flora (ASF) have been shown to exhibit significant decreases in amplitude and frequency of smooth muscle contractions compared to specific-pathogen free (SPF) mice as early as postnatal day 3 (45). Similar to antibiotic-induced dysbiosis, GF and ASF mice exhibit significant reductions in myenteric neurons and disruptions in nerve fibers within the myenteric plexus (45). Conventionalization of GF mice with SPF flora may rescue the excitability of myenteric neurons (46). Together, these findings indicate an important role for the gut microbiome in facilitating intestinal motility beginning in early life.

The mechanisms through which intestinal bacteria mediate motility via the ENS remain unclear. This interaction was traditionally believed to occur through immune mediators (47). However, increasing evidence supports the role of direct microbiome-enteric neuron interactions. Enteric neurons have been shown to directly express TLRs, with knock-down models demonstrating significant reductions in intestinal motility (48–50) and thus exhibiting a constipated phenotype. This suggests that bacteria or bacterial products may interact directly with enteric neurons to facilitate ENS maturation, function, and intestinal motility.

#### The Gut Microbiome of Children With Constipation

To date, five studies have investigated the gut microbiome of children with constipation (51, 52). Zhu and collaborators (28) carried out the first study using 16s rRNA gene pyrosequencing, finding that obese children with constipation consumed less fiber and had lower levels of *Prevotella* and increased levels of butyrate-producing taxa (*Coprococcus, Roseburia*, and *Faecalibacterium*) than controls. The authors hypothesized that increased butyrate production in constipated children may contribute to the development of the disease, which contradicts other preclinical and adult findings in which butyrate exhibited the opposite effect (37, 38, 53, 54) and that the observed microbiome changes might be the result of a lower fiber intake. It should be noted that no metagenomic analyses were performed and the reported butyrate changes were based on extrapolation of data from other studies that used older techniques to characterize the taxa.

In the most recent study (52), the authors used quantitative real-time polymerase chain reaction (qPCR) to evaluate seven species of *Lactobacillus* in the fecal samples of 40 children with FC and 40 controls. Constipated children were found to have lower levels of *Lactobacillus* spp. than controls. However, an important limitation of this study was the omission of an evaluation of dietary intake, which is an important factor influencing the composition of the gut microbiome (55). Similarly, in another study that employed PCR-based analyses, children with constipation were found to have lower levels of *Lactobacillus* spp. than non-constipated children despite a similar intake of fruits and vegetables, although constipated children consumed more dairy products and junk food (29). Thus, the authors concluded that the lower levels of *Lactobacillus* spp. might be the result of diet modulation.

Meij et al. (30) prospectively enrolled children with intractable constipation and analyzed their gut microbiomes by using PCR-based analyses, finding microbiome differences from the healthy control group only after employing a ridge regression model: children with constipation had increased abundance of the phyla Proteobacteria, increased levels of *Bacteroides* spp., *Parabacteroides* spp., and *Bifidobacterium longum*, and decreased levels of *Alistipes finegoldii* and *Ruminococcus* spp. An important limitation of this study, again, was the fact that dietary intake was not evaluated.

Finally, a study from 1998 (56) found that children with FC who followed similar Mediterranean diets exhibited higher levels of the genera *Clostridia* and *Bifidobacteria* than healthy controls. However, important limitations included the use of culture-dependent approaches which carry a risk of missing bacteria that remain difficult to culture under older laboratory conditions and the omission of assessing dietary adherence. Table 1 summarizes the characteristics of studies assessing the role of the gut microbiome on pediatric FC.

**Table 1:** Characteristics of studies assessing the gut microbiota of children with constipation.

**References**	**Year**	**Country**	**Study population**	**Microbiota analysis**	**Dietary assessment**	Laxative treatment^*^	**Findings**
Jomehzadeh et al. (52)	2020	Iran	40 children with FC and 40 healthy controls aged 4–18 years	qPCR	No	No	Lower levels of *Lactobacillus* spp. (*L. casei, L. paracasei, L. rhamnosus, L. plantarum, L. reuteri, L. fermentum, L. acidophilus*) in children with FC children than controls
De Meij et al. (30)	2016	Netherlands	76 children with FC and 60 controls aged 4–18 years	16S rDNA sequencing	No	No	Higher levels of *Bacteroides* spp., *Parabacteroides* spp., *Bifidobacterium longum, Proteus mirabilis*, and lower levels of *Alistipes finegoldii* and *Ruminococcus* spp. than controls
Moraes et al. (29)	2016	Brazil	39 children with FC and 40 controls aged 6 months to 3 years	qPCR	Yes	Not specified	Lower levels of *Lactobacillus* spp, shorter duration of breastfeeding, and higher rates of cesarian section in children with FC than controls
Zhu et al. (28)	2014	USA	8 constipated obese children and 14 controls	16S rRNA pyrosequencing	Yes	Not specified	Lower fiber intake and levels of *Prevotella*, and increased levels of *Coprococcus, Roseburia* and *Faecalibacterium* in children with FC than controls
Zoppi et al. (56)	1998	Italy	28 children with constipation and 14 healthy controls aged 5–14 years	Culture	Yes	No	Higher levels of genera *Clostridia* and *Bifidobacteria* in children with FC than controls

* *Laxative treatment within 4 weeks prior to study*.

#### The Gut Microbiome of Adults With Constipation

The gut microbiome of adults with constipation and slow colonic transit times has also been investigated (57). A 2020 study (54) of adult subjects with long colonic transits – but without constipation – showed that increased α-diversity (i.e., the diversity of taxa within a sample/sampled site) was significantly associated with longer descending colon transit time but not with stool consistency. In addition, longer rectosigmoid transit time was associated with low concentrations of fecal SCFAs (butyrate, propionate, valerate, and caproate), while fecal caproate and plasma acetate were associated with longer transit times of the whole colon and descending colon, respectively. Bacteria belonging to the *Oscillospira* genus may also be implicated in the pathogenesis of constipation in adults (58).

Moreover, Mancabelli et al. (53) conducted a study that employed 16S rRNA sequencing and shotgun metagenomics to study the gut microbiome of adults with and without FC. Non-constipated subjects exhibited significantly higher levels of *Bacteroides, Roseburia* and *Coprococcus*, and lower levels of *Faecalibacterium* than constipated subjects. As the first three are butyrate-producing taxa, the authors hypothesized that the motility-stimulating effect exerted by these SCFAs may “counteract” the development of FC. Furthermore, the metagenomic analysis revealed that non-constipated subjects exhibited a higher abundance of genes involved in carbohydrate and fatty acid metabolism (pathways involved in SCFAs production), while the constipated group had a higher abundance of genes involved in methane production (a gas that delays intestinal transit; see next section). Importantly, both groups consumed similar diets, with non-constipated subjects consuming more sweets, however, this did not reach statistical significance. This is thus far the most informative study as it evaluated the composition and functionality of the gut microbiome of constipated and non-constipated subjects. A further similar study of children would certainly be of great interest.

#### Methanogenic-Type Small Intestinal Bacterial Overgrowth

Methane, a biologically active gas mainly produced by members of the domain Archaea (e.g., *Methanobrevibacter smithii*) as a by-product of carbohydrate fermentation (59), has been shown to delay intestinal transit in animal and human models (60). There is a subgroup of children with methanogenic-type small intestinal bacterial overgrowth (SIBO) who present with constipation (60) and, importantly, may have additional symptomatology that can help to distinguish them from non-methane producers. For example, symptoms such as retentive fecal incontinence, abdominal pain, flatulence, and bloating are more likely to be present in these children (61–63). Methanogenic-SIBO can be diagnosed via the methane/hydrogen breath test and these children may benefit from antibiotic treatment (60).

#### Microbiota-Based Treatments and Functional Constipation in Children

One of the main purposes of understanding the gut microbiome of constipated children is to develop therapeutics such as prebiotics, probiotics, and symbiotics, as well as other microbiome-derived motility-promoting interventions. Thus, it is paramount to first understand the composition and functionality of the gut microbiome in children with constipation. Paradoxically, more research has been conducted on the effects of probiotics than on the gut microbiome itself. The latest systematic review (51) assessed eight randomized placebo-controlled trials (RCTs) which investigated the role of various strains of probiotics on the treatment of childhood FC. Only one RCT showed significant improvement in stool frequency and consistency, and reduction in abdominal pain using *Lactobacillus casei rhamnosus* Lcr35 (64), however, these findings were contradicted by a later trial (65), and the pooled results of the two RCTs showed no statistical significance for treatment success (66). Previous systematic reviews (66–69) have also demonstrated insufficient evidence to recommend the use of prebiotics, probiotics, or synbiotics in the treatment of childhood FC, and highlighted important inconsistencies and methodologic errors in prior systematic reviews (70). Table 2 depicts the characteristics of studies assessing the effects of probiotics on children with constipation.

**Table 2:** Characteristics of studies assessing the effects of probiotics on children with constipation.

**References**	**Year**	**Country**	**Age (yr)**	***n* (intervention/control)**	**Intervention Protocol (intervention, control, duration)**	**Clinical Response Criteria**	**Treatment Effect (intervention vs. control)**
Banaszkiewicz et al. (71)	2005	Poland	2–16	84 (43/41)	*Lactobacillus rhamnosus* GG, + lactulose Placebo + lactulose 12 wk, 24 wk	≥3 spontaneous BM per week, decreased episodes of fecal incontinence	≥3 spontaneous BM frequency NS, 12 wk ≥3 spontaneous BM frequency NS, 24 wk
Bu et al. (64)	2007	Taiwan	<10	45 (18/18/9)	*Lactobacillus casei rhamnosus* Lcr35 Magnesium oxide Placebo 4 wk	≥3 spontaneous BM per week, decreased episodes of fecal incontinence in the fourth week	Achieved ≥3 spontaneous BM frequency (0.57 ± 0.17 vs. 0.55 ± 0.13 vs. 0.37 ± 0.10; *P* = 0.03)
Hannah et al. (72)	2008	Indonesia	0.5–14	43 (23/20)	Synbiotic mixture^a^ Placebo + saccharin 1 wk	Increased BM frequency, improvement in BM consistency, decrease in pain with defecation, no treatment side effects	Increased BM frequency, improved BM consistency, improvement in pain with defecation, side effects NS
Coccorullo et al. (73)	2010	Italy	>0.5	44 (22/22)	*Lactobacillus reuteri* DSM 17938 Placebo 8 wk	≥3 spontaneous BM per week	Achieved ≥3 spontaneous BM frequency (*P* = 0.008)
Khodadad et al. (74)	2010	Iran	4–12	102 (37/33/32)	Synbiotic mixture (Protexin)^b^ + liquid paraffin Synbiotic mixture (Protextin)^b^ + placebo Liquid paraffin + placebo 4 wk	≥3 spontaneous BM per week, ≤ 2 fecal soiling per month, decrease in abdominal pain	Achieved ≥3 spontaneous BM all groups (*P* <0.001, highest in symbiotic + liquid paraffin group), fecal soiling and abdominal pain NS
Guerra et al. (75)	2011	Brazil	5–15	60 (30/30)	Goat yogurt with probiotic cultures^c^ Placebo (goat yogurt without probiotic cultures) 10 wk (crossover at 5 wk)	Increased BM frequency, improvement in BM consistency, decrease in abdominal, decrease in pain with defecation	Increased BM frequency (*P* = 0.012), BM consistency NS, improvement in abdominal pain (*P* = 0.015), improvement in pain with defecation (*P* = 0.046)
Tabbers et al. (76)	2011	Netherlands and Poland	3–16	159 (79/80)	Activia with probiotic cultures^d^ Placebo (yogurt without probiotic cultures + low lactose content) 3 wk	≥3 spontaneous BM per week, <1 fecal incontinence in the last 2 weeks	≥3 spontaneous BM NS
Sadeghzadeh et al. (77)	2014	Iran	4–12	56 (28/28)	Probiotic mixture (Protexin)^e^ + lactulose Placebo + lactulose 4 wk	Increased BM frequency, improvement in BM consistency	Increased BM frequency all groups (2.08 ± 0.65 and 1.54 ± 0.98 *P* = 0.042), improved BM consistency (0.88 ± 0.45 vs. 0.63 ± 0.50, *P* = 0.049)
Hashemi et al.^*^ (78)	2015	Iran	2–16	120 (40/40/40)	Synbiotic mixture (Kidilact)^b^ + PEG Placebo + synbiotic mixture (Kidilact)^b^ Placebo + PEG 6 wk	Not available	Significant improvement in constipation symptoms with synbiotic mixture + PEG.
Abediny et al.^*^ (79)	2016	Iran	4–12	90 (45/45)	Synbiotic mixture (Kidilact)^b^ + PEG PEG 4 wk	Increased BM frequency, decrease in abdominal pain, decrease in painful defecation	BM frequency and painful defecation NS; improved abdominal pain (*P* < 0.05)
Wojtyniak et al. (65)	2017	Poland	<5	94 (48/46)	*Lactobacillus casei rhamnosus* Lcr35 Placebo, 99% milk powder + 1% magnesium stearate 4 wk	≥3 spontaneous BM per week, decreased episodes of fecal incontinence in the last week of intervention	≥3 spontaneous BM NS
Mahdavi et al. (80)	2017	Iran	2–10	94 (48/46)	Synbiotic mixture (Kidilact)^b^ + PEG PEG 4 wk	Increased BM frequency, improvement in stool consistency, decrease in pain with defecation, decrease in number of withholding episodes per week, decreased episodes of fecal incontinence per week, no treatment adverse events	BM frequency, stool consistency, pain, and withholding NS; improvement in fecal incontinence (0.289 vs. 0.02, *P* = 0.005)
Russo et al. (81)	2017	Italy	4–12	55 (27/28)	Probiotic mixture (Tribif)^f^ + PEG PEG 8 wk	≥3 spontaneous BM per week, increased BM frequency, improvement in BM consistency Bristol ≥ grade 3, decrease in abdominal pain, decreased episodes of fecal incontinence, decreased rectal bleeding with defecation	≥3 spontaneous BM, BM frequency, BM consistency, abdominal pain, fecal incontinence and rectal bleeding NS
Bastürk et al. (82)	2017	Turkey	4–18	155 (77/78)	Synbiotic mixture^g^ + prebiotics^h^ Placebo 4 wk	Increased BM frequency, improvement in BM consistency (Bristol type), decrease in abdominal pain, decrease in pain with defecation	Increased BM frequency (*p* ≤ 0.001), improvement in BM consistency (*p* ≤ 0.001), improvement in abdominal pain (*p* ≤ 0.001), improvement in pain with defecation (*p* ≤ 0.001)
Jose et al. (83)	201	India	<18	60 (30/30)	Probiotic mixture (Protexin)^b^ + lactulose Placebo + lactulose 4 wk	Increased BM frequency, improvement in BM consistency, decrease in abdominal pain, decreased episodes of fecal incontinence, no treatment side effects	Increased BM frequency, improvement in BM consistency; abdominal pain and fecal incontinence NS
Wegner et al. (84)	2018	Poland	3–7	129 (65/64)	*Lactobacillus reuteri* DSM 17938 + PEG Placebo + PEG 8 wk	≥3 spontaneous BM per week, increased BM frequency, improvement in BM consistency, decrease in pain with defecation, decreased episodes of fecal incontinence	≥3 spontaneous BM, BM frequency, BM consistency, painful defecation and fecal incontinence NS
Jadrešin et al. (95)	2018	Croatia	2–18	33 (18/15)	*L. reuteri* DSM 17938 + lactulose Placebo + lactulose 12 wk	Increased BM frequency, improvement in BM consistency, improvement in symptoms at the end of the study, decreased dose of the lactulose needed, and decreased episodes of fecal incontinence	BM frequency, BM consistency, presence of symptoms, lactulose and fecal incontinence NS

*Data extracted from systematic review and meta-analysis by Harris et al. (70) BM, bowel movement; NS, not significant; PEG, polyethylene glycol; RR, relative risk*.

a *Lactobacillus acidophilus, Lactobacillus rhamnosus, Lactobacillus casei, Lactobacillus bulgaricus, Bifidobacterium infantis, Bifidobacterium breve, Streptococcus thermophiles*.

b *Lactobacillus casei, Lactobacillus rhamnosus, Streptococcus thermophilus, Bifidobacterium breve, Lactobacillus acidophilus, Bifidobacterium infantis, fructooligosaccharide*.

c *Bifidobacterium longum, Lactobacillus delbrueckii subsp. bulgaricus, Streptococcus thermophiles*.

d *Bifidobacterium lactis DN-173010, Lactobacillus delbrueckii subsp. bulgaricus, Streptococcus thermophiles*.

e *Lactobacillus casei PXN 37, Lactobacillus rhamnosus PXN 54, Streptococcus thermophiles PXN 66, Bifidobacterium breve PXN 25, Lactobacillus acidophilus PXN 35, Bifidobacterium infantis (child specific) PXN 27, and Lactobacillus bulgaricus PXN 39*.

f *Bifidobacterium breve M-16 V, Bifidobacterium infantis M-63, Bifidobacterium longum BB536*.

g *Lactobacillus casei, Lactobacillus rhamnosus, Lactobacillus plantarum, Bifidobacterium lactis*.

h *Fiber, polydextrose, fructo-oligosaccharides, galacto-oligosaccharides*.

* *Full text not available in English*.

An alternative microbiota-based therapy that is gaining increasing popularity for the treatment of FC is fecal microbiota transplantation (FMT). To date, only one adult RCT (85) has been published and showed a 30% higher cure rate compared to conventional treatment with laxatives, probiotics, and behavioral training (85). Additional pilot studies demonstrated similar improvements in symptoms and quality of life (86–88) and successful engraftment of donor microbiota (89), with benefits lasting for at least 1-year post-treatment (88, 89). No clinical trials have yet been published assessing the role of FMT in the treatment of pediatric FC, however, three trials are currently underway (90–92).

## Discussion, Conclusion, and Future Directions

Based on preclinical and clinical evidence, disturbances in the gut microbiome may alter intestinal physiology and motility, and contribute to the development of constipation. However, it is important to mention that the currently available evidence is limited by a paucity of pediatric studies and varying study methodologies. It appears that SCFAs, particularly butyrate, can have a motility promoting effect in both human and animal models, although the evidence is controversial. The role of other molecules in FC, such as 5-HT, bile acids, tryptamine, and GLP-1, warrants further investigation in human models. Furthermore, diet may be a potential confounder as it can modulate gut bacteria, making it difficult to ascertain causal relationships between the gut microbiome and FC. To date, a gut microbiota signature in children with FC has not been identified.

The pathophysiology of FC is multifactorial, encompassing a complex interplay between psychological factors, gut microbiota, and gastrointestinal motility. Disruptions to the gut microbiome may contribute to the development and outcome of FC in children. More research is required to elucidate the mechanisms through which the gut microbiome influences intestinal motility and to assess the role of microbiota-based interventions in pediatric FC.

We believe that future studies assessing the gut microbiome in children with FC should: (1) include constipated (withholders and non-withholders) children fulfilling the Rome IV criteria and a control group (93); (2) ensure children are off laxatives at the time of enrollment; (3) evaluate dietary intake as accurately as possible (before [recall] and during the study) (94), as well as early-life gut microbiome insults variables; and, (4) to assess the gut microbiota via, ideally, 16S rRNA sequencing, metagenomic shotgun sequencing, and transcriptomics analyses, at the time of enrollment (off-laxative) and then while on treatment (multiple samples) (94).

## Author Contributions

DA contributed conception and design of the manuscript. DA and JP wrote the first draft of the manuscript. ET and ER wrote and edited sections of the manuscript. All the authors contributed to manuscript revision, read, and approved the submitted version.

## Conflict of Interest

The authors declare that the research was conducted in the absence of any commercial or financial relationships that could be construed as a potential conflict of interest.
